# Effects of Functional Coatings Containing Chitosan, Orange Peel and Olive Cake Extracts on the Quality Attributes of Cucumber during Cold Storage

**DOI:** 10.3390/plants11141895

**Published:** 2022-07-21

**Authors:** Kashif Ghafoor, Fahad Y. Al-Juhaimi, Isam A. Mohamed Ahmed, Elfadil E. Babiker, Syed Ali Shahzad, Omer N. Alsawmahi

**Affiliations:** Department of Food Science and Nutrition, College of Food and Agricultural Sciences, King Saud University, P.O. Box 2460, Riyadh 11451, Saudi Arabia; faljuhaimi@ksu.edu.sa (F.Y.A.-J.); iali@ksu.edu.sa (I.A.M.A.); ebabiker.c@ksu.edu.sa (E.E.B.); syedalishahzad@gmail.com (S.A.S.); omernasser2009@hotmail.com (O.N.A.)

**Keywords:** cucumber, functional coating, chitosan olive cake extract, orange peel extract, physicochemical quality, shelf-life

## Abstract

This study investigated the effect of functional coating using 2% chitosan and different concentrations of olive cake extract (OCE) and orange peel extract (OPE) on the physicochemical quality attributes of cucumber during cold storage at 4 °C for 21 days. Both coating and storage influenced (*p* ≤ 0.05) the physicochemical attributes of cucumber. The highest values of moisture content, total soluble solids (TSS), pH, total phenolic contents (TPC), DPPH radical scavenging activity, yellowness (b*), and hardness were found in coated samples, which also showed the lowest values of the lightness (L*), greenness (a*), total viable count (TVC), yeast and mold counts, and acidity (*p* ≤ 0.05). Uncoated cucumber samples showed the highest (*p* ≤ 0.05) levels of acidity, lightness, greenness, TVC, and yeast and mold count. During storage, concomitant (*p* ≤ 0.05) reduction in moisture, TSS, pH, TPC, DPPH radical scavenging activity, L*, a*, b*, and hardness along with concurrent (*p* ≤ 0.05) increment in acidity, TVC, and yeast and mold count were evident in all cucumber samples. Interestingly, the changes in the aforementioned attributes were minimal in functionally coated samples in comparison to uncoated ones, suggesting the potential of OCE and OPE to preserve quality attributes of cucumber during cold storage.

## 1. Introduction

Cucumber (*Cucumis sativus* L.) is one of the most important commercial vegetable crops cultivated in all regions worldwide with an annual production of 91.3 million tons in 2020 [1]. Nutritionally, cucumber contains a high amount of water, low calories, high levels of phenolic compounds and cucurbitacins, and possesses antioxidant, antidiabetic, anti-inflammatory, anti-hyperglycemic, and anti-carcinogenic activities [2,3]. However, this important vegetable product is very perishable, and during postharvest handling and processing, its quality attributes are easily deteriorated [4]. Postharvest water and firmness losses, microbial decay, discoloration, chilling injury, and shriveling result in the very short shelf-life (<14 days) of cucumber [5]. Elongation of the shelf-life of cucumber by different means is very essential for producers, traders, distributors, and consumers, which can reduce the losses and costs, and save the quality of this important product. In this regard, several preservation methods, such as chemical (pesticides and fungicides preservatives), physical (modified and controlled atmospheric packaging, cold storage, and minimal processing), and innovative (edible coating) methods have been used to extend the shelf-life of cucumber [6,7]. Among these approaches, chemical and physical methods are costly, non-environmentally friendly, non-biodegradable, and harmful to human health [6]. An edible coating overcomes the drawbacks of other preservation methods of cucumber and is considered a natural, environmentally friendly, and cheap method [8]. In this process, the coating films act as a barrier on the surface of coated products, modifying the atmospheric gas composition, reducing moisture and weight losses, and delaying senescence and ripening processes, thereby elongating the shelf-life of fresh fruits and vegetables [9]. Another advantage of an edible coating is that essential functional and active ingredients could be incorporated into the edible film and thus can improve the nutritional, sensorial, and health quality attributes of the products [8]. Edible coating for fruits and vegetables can be made with various natural materials, having film-forming properties, including cellulose, starch, natural gums and chitosan [8,10]. Among these materials, chitosan and its derivatives are by far the most frequently used materials in the edible coating of fruits and vegetables due to their abundance, inherent biocompatibility, biodegradability, low cost, superior film-forming potentials, and ability to incorporate natural antimicrobial and antioxidant ingredients [11]. To date, cucumber has been treated with different types of edible coating using various types of chitosan-based [7,12,13,14,15,16], gum-based [4,6,17], starch-based [18,19] and cellulose-based [5] edible coatings, with or without active ingredients from natural sources, and has been found to improve the storage stability of the product. Olive cake and orange peel are the major byproducts of olive oil and orange juice industries, and these underutilized byproducts are considered an essential source of phytochemicals with high antioxidant and antimicrobial activities [20,21,22]. Nonetheless, there is no report on the use of edible coating of cucumber with olive cake extract (OCE) and orange peel extract (OPE). Therefore, this study was conducted to investigate the effect of an edible coating with chitosan containing different concentrations of OCE and OPE on the physicochemical quality attributes of cucumber during cold storage at 4 °C for 21 days.

## 2. Results and Discussion

### 2.1. Effect of Coating and Cold Storage on the Moisture Content, Water Activity, and Total Soluble Solids of Cucumber

The results on the moisture content, water activity (aw), and total soluble solids (TSS) of cucumber as affected by coating and storage are shown in Table 1. Irrespective of the treatment and storage duration, high moisture content (>90%) was observed in all cucumber samples. The coating treatments preserve the moisture content of cucumber; higher moisture content was found in coated cucumber samples compared to uncoated samples (*p* ≤ 0.05). However, prolonged storage significantly (*p* ≤ 0.05) reduced the moisture content to the minimum values at day 21 of storage of all samples. Improved moisture retention of coated cucumber samples is likely because the chitosan coating with or without functional ingredients acts as barrier against water evaporation, suppressing transpiration [14]. The reduction in moisture content during prolonged storage could be attributed to the evaporation and transpiration processes that usually occur during the storage of fruits and vegetables due to variations in temperature and relative humidity [6]. In agreement with our findings, it has been reported that coating treatment reduced moisture loss during the storage of cucumber at a low temperature [23]. In addition, increased weight losses in fruits and vegetables during storage was attributed to moisture losses, and various studies indicated that coating treatments reduced the weight loss of cucumber samples during storage under different conditions [6,13,14,15], which is in accordance with our findings.

The water activity of the cucumber samples was not influenced by both coating treatments and storage duration; however, it showed insignificant reduction during cold storage. The water activity of all cucumber samples stored at 4 °C was above 0.960, suggesting the susceptibility of cucumber to spoilage by mesophilic microbes if not well preserved. The decline of water activity during storage could be due to the reduction in moisture during the cold storage of cucumber. It is well known that fresh cucumber is susceptible to decay and spoilage by microorganisms, as it has a high moisture content (95%) and water activity (0.96) and consequently requires special processing treatments and preservation conditions [24].

The TSS increased significantly (*p* ≤ 0.05) in cucumber coated with different concentrations of OCE or OPE, compared to the untreated control or samples coated with 2% chitosan only. The lowest TSS value was seen in untreated control samples stored for 21 days, whereas the highest value of TSS was observed in fresh cucumber samples treated with OCE and OPE, suggesting that functional ingredients in OCE and OPE improved the TSS of cucumber samples. The increased TSS in functionally coated cucumber could be due to the presence to reducing sugars in OCE and OPE used in the coating process of cucumber. In addition, coating cucumber with chitosan containing OCE and OPE might reduce the respiration rate, thereby maintaining higher TSS compared to uncoated samples. Similarly, previous reports indicated that cucumber coated with chitosan containing salicylic acid [25] and carbon dots [15] as functional ingredients possessed higher TSS than uncoated samples. During storage, a concomitant reduction (*p* ≤ 0.05) in TSS was observed as the storage time increased to 21 days, reaching the minimum values at the end of storage of all samples. The reduction in TSS during cold storage is likely due to the senescence of the cucumber samples [15].

### 2.2. Effect of Coating and Cold Storage on the pH, Acidity, and Bioactive Properties of Cucumber

The results on the pH, acidity, total phenolic content (TPC), and antiradical activity (DPPH radical inhibition activity of cucumber samples coated with 2% chitosan with or without functional ingredients and stored at 4 °C for 21 days are presented in Table 2. The pH of cucumber was influenced by the coating treatment and storage time at different magnitudes (*p* ≤ 0.05). Coating generally increased the pH of fresh and stored cucumber samples, and the highest values were observed in sample F that was coated with 2% chitosan containing 2% OPE, whereas the lowest values were observed in untreated control sample (A) during cold storage (*p* ≤ 0.05). Similarly, high pH values were observed in cucumber coated with modified corn starch/gelatin films [26] and latex coating with calcium oxide nanoparticles [27]. During storage, the pH of untreated control sample (A) was simultaneously reduced as the storage period progressed, whereas that of the coated cucumber samples increased to the maximum values at day 14 of storage and then reduced again as the storage time increased. The reduction in pH of uncoated cucumber during storage could be due to the metabolic conversion of sugars into acids [7]. The increase in pH of coated cucumber during storage is likely due to the utilization of organic acids in the respiration metabolism, and the following reduction in pH at elongated storage is probably due to the conversion of sugars into organic acids [5]. Similar observations on the increase in pH in fresh and stored coated cucumber have been reported in numerous reports [5,7,18,26].

The acidity of the cucumber samples was affected by the coating treatment and storage duration (*p* ≤ 0.05). Generally, functional coating reduced the acidity of the cucumber samples, and the minimum values were seen in the samples coated with 2% OPE which remained the lowest during the storage of all samples, whereas the highest values were observed in the uncoated samples (*p* ≤ 0.05). The reduction in acidity following the coating treatment is likely due to the utilization of organic acids in respiration processes. During storage, the acidity of the cucumber samples was concomitantly (*p* ≤ 0.05) increased as the storage time elongated, and the highest values were recorded for samples stored for 21 days at 4 °C; however, the lowest increasing trend was seen in the coated cucumber samples. The increase in acidity during storage could be attributed to the conversion of sugars into organic acids [5]. As loss of vegetable acidity has been linked to the decline of the quality of vegetables during storage, the retention of acidity in the coated cucumber samples is beneficial in preserving the quality and elongating the shelf-life of the products [6]. Previous reports indicated that both coating and storage conditions affected the titratable acidity of cucumber in different ways [5,6,18,27].

As shown in Table 2, differences in TPC were observed between the control and coated samples, in which the lowest TPC was evident in the uncoated cucumber samples, whereas the highest values were found in samples coated with chitosan containing different concentrations of functional ingredients (*p* ≤ 0.05). The increase in TPC of cucumber samples coated with functional ingredients (OCE and OPE) is likely due to the richness of these natural byproducts (olive cake and orange peel) with phenolic compounds [20,21]. During storage, the TPC of all cucumber samples showed concurrent (*p* ≤ 0.05) reduction as the storage period elongated, reaching the minimum values at the end of storage (21 days). In spite of the reduction in TPC during storage, its levels in uncoated cucumber samples remained the lowest, whereas those of the coated samples containing (2% OPE) remained the highest at all storage intervals (*p* ≤ 0.05). The reduction in TPC during storage is likely due to the utilization of these important phytochemicals as preservatives and antioxidant agents against microbial growth and oxidation of cucumber constituents. Hence, coating cucumber with functional ingredients from olive cake and orange peel could significantly improve the storage stability of this perishable product. The findings of this study are comparable to those of previous reports, which indicated that coated cucumber possessed higher total phenolic contents than uncoated samples, and it was reduced in a lesser manner during storage, compared to that of the uncoated samples [6,27].

In accordance with TPC, the DPPH antiradical activity of uncoated cucumber was lower than that of samples coated with chitosan containing different concentrations (1% and 2%) of OCE or OPE (Table 2). The highest DPPH radical scavenging activity was seen in cucumber samples coated with 2% OPE, followed by that coated with 1% OPE, 2%OCE, and then 1% OCE (*p* ≤ 0.05), indicating that the functional ingredients used in coating cucumber samples improved the functional properties of cucumber samples [6,27]. During storage, the antioxidant activity of all samples continually increased to the maximum values at day 14 of storage and then reduced again as the storage time progressed (*p* ≤ 0.05). It is worth noting that the DPPH radical scavenging activity of the cucumber sample coated with 2% OPE was 2 times higher than that of the untreated control at all the storage intervals, suggesting the antioxidative potentials of this functional material in coated cucumber samples, and hence could be used to enhance the nutritional and health quality and to extend the shelf life of cucumber [6,27]. In agreement with our findings, previous reports demonstrated that the coating treatment improved the antioxidant activity of fruits during storage [6,27,28,29]. Overall, the functional coating treatment of cucumber using chitosan containing olive cake extract and orange peel extract could improve the storage stability and health quality attributes of the product.

### 2.3. Effect of Coating and Cold Storage on the Microbial Load of Cucumber

**Figure 1:**
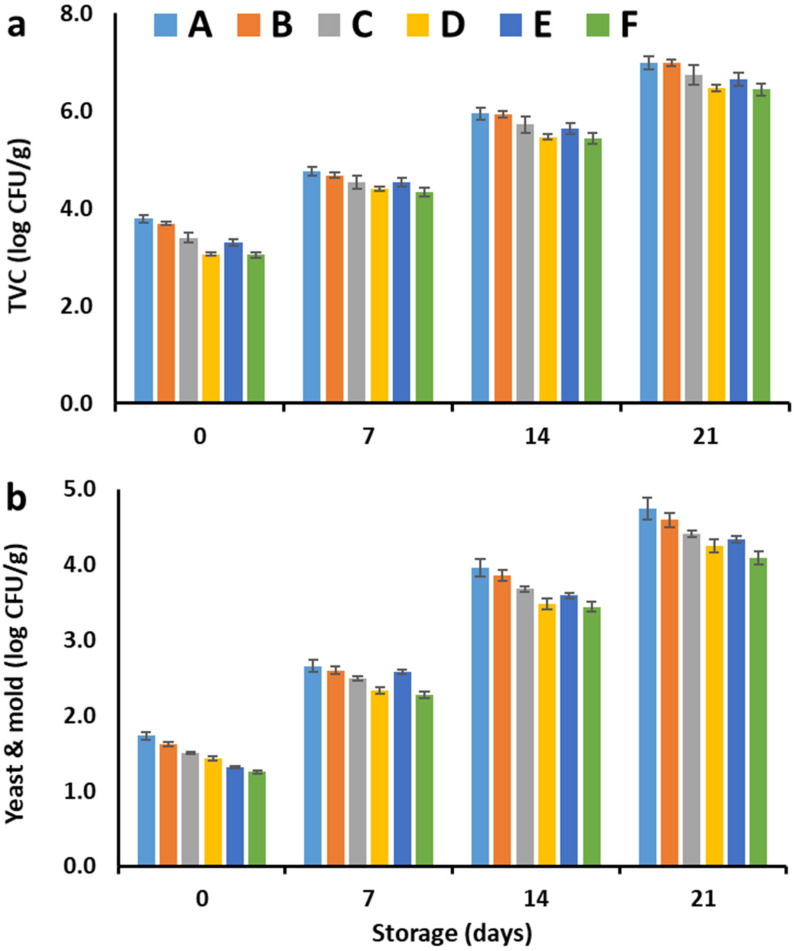
Changes in (**a**) total viable count (TVC) and (**b**) yeast & mold count of fresh cucumber coated with chitosan supplemented with different concentrations of olive cake extract (OCE) or orange peel extract (OPE) during cold storage (4 °C). A: Uncoated, B: Coated with 2% chitosan only, C: Coated with 2% chitosan containing 1% OCE, D: Coated with 2% chitosan containing 2% OCE, E: Coated with 2% chitosan containing 1% OPE, and F: Coated with 2% chitosan containing 2% OPE.

Figure 1 demonstrates the variation in the total viable count (TVC) and yeast and mold counts of cucumber samples as affected by the coating treatment and storage duration. TVC (Figure 1a) and yeast and mold counts (Figure 1b) were the highest in the uncoated cucumber samples followed by those coated with 2% chitosan only, whereas the lowest counts were seen in cucumber coated with functional materials (2% OCE and 2% OPE), suggesting the protective effects of these materials against spoilage microbes in cucumber samples. The reduction in TVC and mold and yeast counts in the coated cucumber with chitosan containing OCE and OPE is likely due to the presence of the phytochemicals with high antimicrobial activity of olive cake and orange peel extract as reported previously [22,30]. In addition, the coating material formed a protective layer on the surface of the cucumber samples, thereby reducing the microbial contamination and growth [9]. As the storage time progressed, the TVC and yeast and mold counts of all samples were continually (*p* ≤ 0.05) increased to the maximum values at the end of storage. Cucumber samples coated with 2% OCE and 2% OPE showed the lowest counts, whereas the uncoated samples showed the highest counts at all storage intervals indicating the protective effects of the used coating materials during cold storage (4 °C). A food product carrying less than 6 log CFU is generally considered safe, and the results presented in Figure 1 indicate that all OPE- and OCE-chitosan coated samples carried a TVC count less than this value for up to 14 days of refrigerated storage. The TVC count became higher than 6 log CFU in both uncoated and chitosan coated samples, indicating the potential of the antimicrobial effects of OPE and OCE [22]. In agreement with these findings, previous reports demonstrated the edible coating of cucumber with carbon dots and chitosan [15], limonene and chitosan [7], guar gum [6], chitosan nanoparticles loaded with Cinnamomum zeylanicum essential oil [13], aloe vera gel and carboxymethyl cellulose composite [5], and saffron petal extract containing konjac glucomannan edible films [17].

### 2.4. Effect of Coating and Cold Storage on the Surface Color of Cucumber

Table 3 shows the changes of surface color attributes (L*; lightness, a*; greenness, and b*; yellowness) of uncoated and coated cucumber samples during cold storage. The coating treatment and storage time affected the color attributes in different ways (*p* ≤ 0.05). The lightness values of uncoated cucumber were the highest at all storage intervals, which were reduced to the minimum values in cucumber coated with 1% OCE and then increased again for the samples coated with 2% OPE. The reduced lightness of coated cucumber could be attributed to the opacity and dark color of the functional coating materials [27]. During storage, the lightness values of all cucumber samples were reduced to minimum values at the end of the storage period. Similarly, previous reports showed lower in L* values in coated fruits, compared to uncoated ones, and attributed that to the opacity and color of coating materials [13,27]. In addition, a reduction in L* values of coated and coated fruits were observed during storage at different temperature and durations [5,13,27].

The greenness (a*) values of cucumber were influenced by both coating treatment and storage duration in different magnitudes (*p* ≤ 0.05). In fresh cucumber, the greenness of uncoated samples was higher than those of coated ones, and greenness values generally reduced upon the coating of cucumbers with different concentrations of OCE and OPE. The reduction in greenness of cucumber following coating with functional materials could be attributed to the dark color of the added ingredients. Compared to fresh samples, prolong storage (21 days) greatly reduced the greenness values of cucumbers; however, the changes in greenness color were minimal in coated samples, suggesting the green color stabilizing effects of coating materials used in this study. The reduction in green color during storage could be due to the enzymatic decomposition of chlorophyll. The coating maintains the green color of cucumber by changing the surface environment of the samples, thereby preventing oxidative and enzymatic browning [13]. In agreement with our findings, previous reports demonstrated a reduction in the greenness of coated and uncoated cucumber during storage, with minor changes in the coated samples compared to the uncoated ones [5,7,13].

The yellowness (b*) values of cucumber were affected by both functional coating and storage duration in fluctuated ways. In fresh cucumber, the highest yellowness value was seen in the uncoated sample, which reduced to the minimum in samples coated with 1% OCE (*p* ≤ 0.05). In stored cucumber, the highest yellowness values were observed in cucumber coated with OPE, whereas the lowest values were seen in uncoated samples. The increase in yellowness of OPE-coated cucumber could be due to the yellow color of orange peel extract that might be released by microorganisms, thereby increasing the surface yellowness of the coated cucumber during storage. Similar changes in yellowness values of coated and uncoated fruits during storage were also reported [5,7,13]. Overall, the functional coating of cucumber with chitosan containing different concentrations of OCE and OPE has positive effects on the color attributes and hence could improve the consumer acceptability of the product [5,7].

### 2.5. Effect of Coating and Cold Storage on the Texture Profile of Cucumber

The changes in the texture profile of cucumber as affected by functional coating and cold storage are shown in Table 4. The hardness, cohesiveness, and springiness of cucumber samples were affected by both the coating treatment and cold storage. The cohesiveness and springiness were slightly affected by the coating and storage treatments. In fresh samples, the highest hardness was observed in the cucumber samples coated with 2% chitosan only followed by those coated with 2% OPE, whereas the lowest values were found in the samples coated with OCE followed by the uncoated cucumber samples (*p* ≤ 0.05). The hardness of all samples concomitantly (*p* ≤ 0.05) decreased following the increase in storage time, reaching minimum values at the end of the storage; however, functionally coated cucumber exhibited higher hardness than uncoated ones, suggesting that functional coating delayed the decay of cucumber during prolonged storage at 4 °C. Generally, coating alleviates the negative effects of storage on the texture of cucumber due to the fact that coating materials form a barrier which reduces the O_2_ availability and metabolic rate, thereby delaying the softening process of coated samples during prolonged storage [15]. Hardness or firmness is a vital factor that determines the marketability and consumer acceptability of fresh fruits and vegetables [5]. The reduction in hardness during storage could be due to the increased rate of senescence and metabolic softening process [15]. In agreement with our findings, a previous report showed a reduction in hardness during the storage of coated and uncoated cucumber; however, the reduction rate in coated samples was lower than that in uncoated ones [5,7,13,15]. The textural and other quality characteristics of cucumber can also be significantly affected by changes in enzyme activity. Generally, enzymes and the antioxidant defense system protect the plant cell walls and hence the overall textural and other quality attributes of fruits and vegetables. Different enzymes, including peroxidase, ascorbate peroxidase and catalase, play an important role during the storage of cucumber [31,32]. Hence, due to this significance, the future studies may include measurements of enzyme activity in response to different coating treatments containing OCE and OPE.

### 2.6. Multivariate Analysis

**Figure 2:**
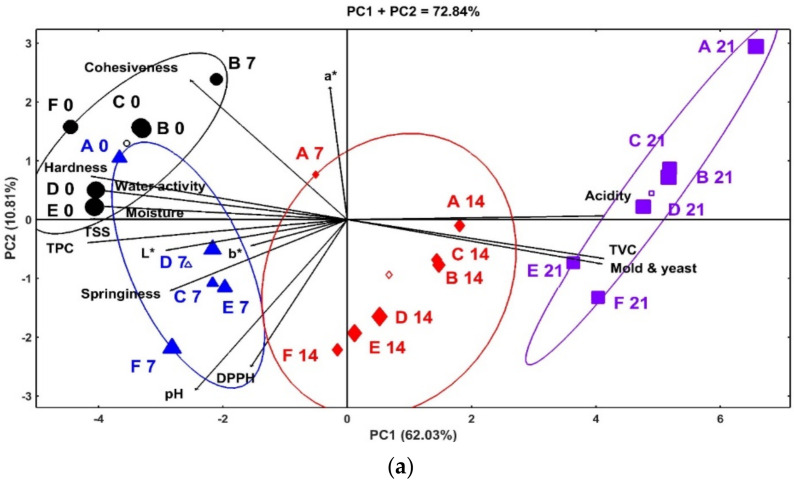

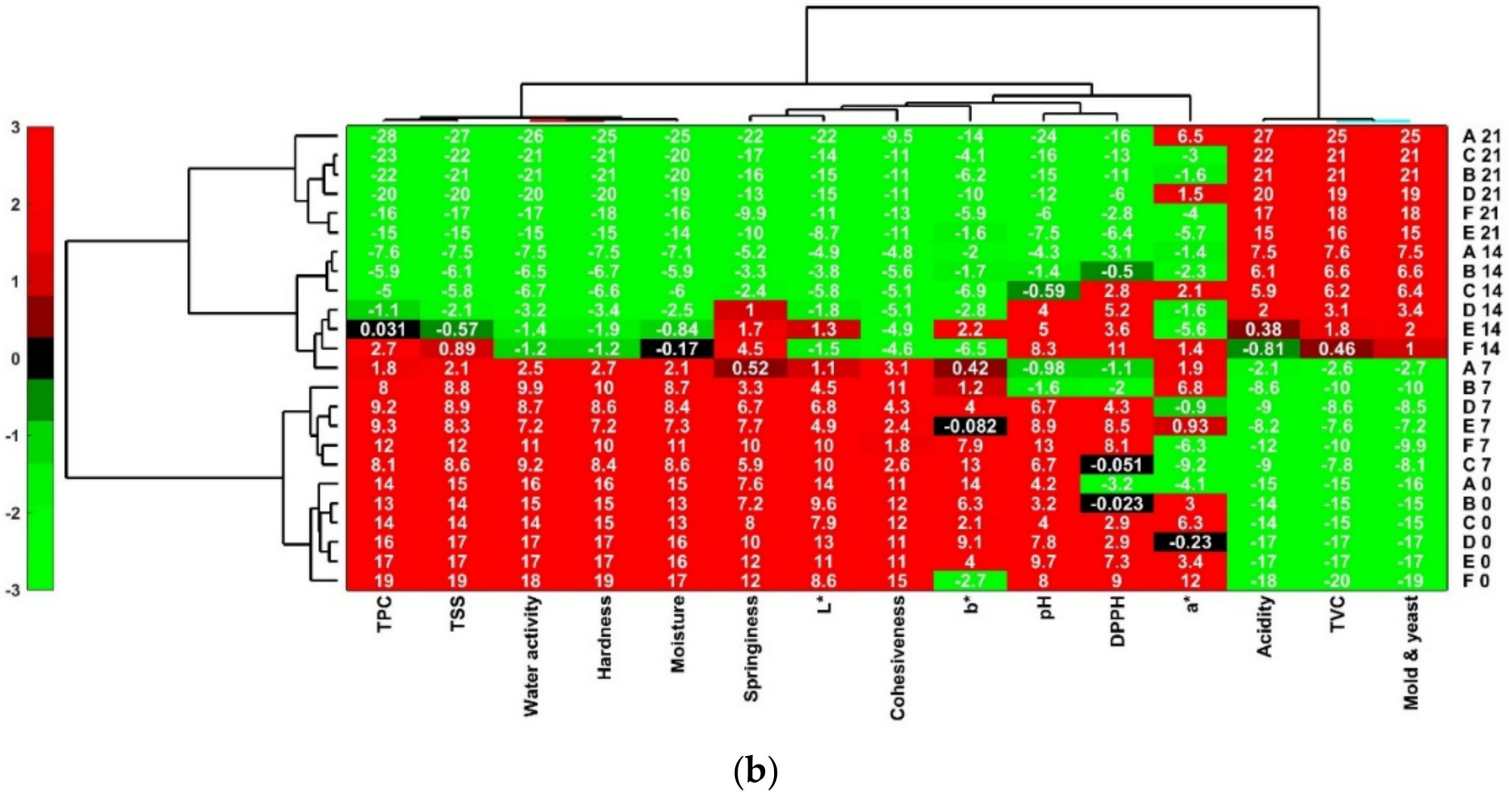
HJ-biplot (**a**) and hierarchical clustering heatmap (**b**) of fresh cucumber coated with chitosan supplemented with different concentrations of olive cake extract (OCE) or orange peel extract (OPE) during cold storage (4 °C) for 0–21 days. A: Uncoated, B: Coated with 2% chitosan only, C: Coated with 2% chitosan containing 1% OCE, D: Coated with 2% chitosan containing 2% OCE, E: Coated with 2% chitosan containing 1% OPE, and F: Coated with 2% chitosan containing 2% OPE. 0, 7, 14, and 21 indicates storage intervals.

Chemometric analysis was conducted by using the principal component analysis (PCA) and hierarchical clustering analysis (HCA) to intensely determine the combined effect of coating with different concentrations of orange peel extract (OPE) and olive cake extract (OCE) and storage time on the physicochemical properties of cucumber (Figure 2). The PCA biplot indicates good contribution of the principal components (PC1, 62.03% and PC, 10.81%) to the overall variability (72.84%) of the plotted data (Figure 2a). In the biplot, the correlations between the traits are verified by the cosine of the angle between the vectors of the traits, in which positive, negative and no correlations are indicated by acute (<90°), obtuse or straight (>90° or 180°) and right (90°) angles, respectively [33]. In this regard, positive correlation was observed between the acidity and microbial load (TVC and yeast/mold), indicating that an increase in microbial counts can increase the acidity of the cucumber samples during storage. The aforementioned attributes were correlated negatively with all other assessed attributes. The increase in acidity of cucumber during storage may contribute to increasing the shelf life, as the loss of acidity of cucumber during storage is reported to affect the quality and storability of coated and uncoated cucumber. Positive correlations were also observed between moisture content, total soluble solids, TPC, water activity, texture attributes, and color attributes, and between the pH and DPPH, suggesting the influence of these attributes on each other. Hierarchical clustering analysis showed four distinct clusters of the treatments based on their impact on the quality attributes of cucumber samples during cold storage (Figure 2a). It is interesting to note that the groups were separated based on the storage effect rather than the treatment effect. The first cluster (right of the graph, purple squire symbol) is composed of uncoated and coated cucumber samples stored for 21 days. This group is characterized by higher levels of acidity and microbial load compared to other groups. Within this group, the highest values of acidity, TVC, and yeast and mold were seen in the untreated control (A21) and chitosan-coated (B21) cucumber samples. The second cluster (middle of the graph, red diamond symbols) is composed of all treated cucumber samples stored at 4 °C for 14 days, except uncoated control (A7) stored for 7 days, with a moderate contribution to acidity and microbial load of cucumber samples. The third cluster (lower left of the graph, blue triangle symbols) contains the coated cucumber samples fortified with different levels of OCE and OPE during 7 days of cold storage, with few exceptions (A0s). This group is characterized by higher levels of color attributes (a* and b*), pH, and DPPH than other groups. The last group (upper left of the graph, black circle symbol) contains all the samples at the beginning of storage and the chitosan-coated sample at day 7 of storage; it is characterized by high levels of texture attributes, moisture content, water activity, TSS and TPC compared to other groups. Hierarchical clustering heatmap (Figure 2b) showed an overall view of the interaction between the treatments and storage time based on their effect on quality attributes of cucumber samples. In the heatmap, the red color indicates high values, whereas the green color indicates low values. Compared to coated samples, the uncoated cucumber samples (A0) showed high values of greenness and brightness colors at the beginning of the storage, whereas, at the end of the storage (A21), it possessed the highest levels of acidity and microbial counts and lowest levels of greenness and brightness color attributes, suggesting the lower storage stability of uncoated cucumber. Functionally coated cucumber samples (C0, D0, E0, and F0) showed highest values of bioactive properties (TPC and DPPH), TSS, moisture, water activity, and textural attributes (hardness, cohesiveness, and springiness) at the beginning of storage and the changes in these attributes during prolonged storage (C21, D21, E21, and F21) were minimal, indicating the improved stability and nutritional and health quality attributes of these samples. Overall, these findings demonstrated that both coating treatment and cold storage duration affected the physicochemical, nutritional, and health quality of cucumber at varied magnitudes. Functional coating improved the physicochemical, nutritional, and health quality attributes and storage stability of cucumber.

## 3. Materials and Methods

### 3.1. Materials

Fresh cucumber samples were obtained from a farm in Riyadh province during the harvesting season (March and April 2022). The samples were transferred to the Laboratory of Food Technology at the Department of Food Science and Nutrition, King Saud University under cooling conditions. Soon after arrival to the Laboratory, the samples were carefully checked, and damaged cucumber and foreign materials were removed. Cucumber samples of regular shape and same size were selected and washed using distilled water to remove dust. Kitchen paper towels were used to gently wipe off water from cucumber surfaces prior to coating experiments. Fresh orange was obtained from a local farm and peeled manually to obtain fresh orange peels, which were dried oven at 45 °C until obtaining constant weight (final moisture was 7.67 ± 1.85%). Fresh olives were acquired from a local market, and the cold press technique was used for oil extraction from the samples using a SUS 304 oil extractor (SUS Machines, Shanghai, China). The olive cake was dried (moisture content 11.32 ± 1.43%). Both the dried olive cake and orange peel were ground into fine powders and kept in sealed bags at 4 °C for further use. All chemicals used were of analytical grade and were obtained from Sigma-Aldrich (Sigma, St. Louis, MO, USA).

### 3.2. Preparation of Olive Cake Extract (OCE) and Orange Peel Extract (OPE)

The preparation of OCE and PCE was carried out as described previously [34]. Briefly, 50 g of olive cake powder or orange peel powder were subjected to 30 min extraction using ddH_2_O at 70 °C. After that, the extract was cooled to room temperature, filtered using Whatman filter paper #1 thimbles (Whatman PLC, Maidstone, UK), and the filtrates were collected. The extraction process of the residues was repeated thrice, and the filtrates were collected, combined and dried at 50 °C using a rotary evaporator. The obtained OCE and OPE were ground and stored in sealed bags at −20 °C for further use.

### 3.3. Preparation of Functional Coating Solution

The chitosan solution containing functional ingredients was prepared freshly as described in our recent study [34]. In brief, a 2% chitosan stock solution was prepared by dissolving 40 g edible chitosan in 2 L of a mixture of 1% acetic acid and 1% glycerol. After that, OCE and OPE powders were added to portions (100 mL each) of the chitosan solution at concentrations of 1% and 2% (*w*/*v*), thoroughly mixed, and then homogenized for 5 min using an Acapulco 30564 kitchen blender (Palson Co., Kunshan, China) until obtaining a smooth solution. The prepared chitosan solutions with or without functional ingredients were used for the coating of cucumber samples as described below.

### 3.4. Functional Coating of Cucumber Samples

For functional coating, six samples (blocks) were prepared as follows: A (uncoated, negative control), B (coated with chitosan (2%) only, positive control), C (coated with chitosan (2%) containing 1% OCE), D (coated with chitosan (2%) containing 2% OCE), E (coated with chitosan (2%) containing 1% OPE), and F (coated with chitosan (2%) containing 2% OPE). Fresh and cleaned cucumber samples were dipped into the above coating solutions for 5 min, carefully removed from the dipping solutions, and dried on the stand sieves for 2–3 h at room temperature with the help of a CK2215 plastic fan (Clickon, Liwan, Guangzhou, China) for the quick and complete drying of coated cucumber samples. After that, cucumber samples were packed in perforated (6 holes on the lid) plastic containers and stored in the refrigerator (4 °C) for up to 21 days. The entire blocks were replicated three times and at 7-day intervals, the cucumber samples were analyzed for physicochemical quality attributes.

### 3.5. Physicochemical Analysis

The moisture content, total soluble solids, and treatable acidity were determined using the standard official method [35]. The moisture was determined using the oven-drying method at 105 °C until a constant weight was obtained. A digital refractometer (DR 6000, A. Kruss Optronic GmbH, Hamburg, Germany) was used to determine the total soluble solids (TSS) of the cucumber samples (°Brix) at 20 °C and the dilution factor was used for the calculation of the °Brix of the samples. The acidity was determined for the cucumber slurry using a titration method using 0.1 N NaOH at pH 8.1. The water activity of the cucumber samples was determined using a CX3-TE Aqualab meter (Labo-Scientifica, Parma, Italy). The pH of the homogenized cucumber samples (5 g in 50 mL distilled water) was measured using a Corning 240 pH meter (Corning Scientific Products, New York, NY, USA).

### 3.6. Color and Texture Analysis

The surface CIE color attributes (L*; lightness, a*; redness, and b*; yellowness) of the cucumber samples during storage were determined using a CR-300 Minolta colorimeter (Minolta Camera Co., Osaka, Japan). The colorimeter was first calibrated using a white plate, and then three readings were taken from different areas of each sample. Texture profile analyses of cucumber samples at different storage intervals (0, 7, 14, and 21 days) were measured using a Brookfield CT3 texture analyzer (Brookfield, Middleboro, MA, USA) as described in the instruction manual. A two-cycle test was used for triplicate analyses of the textural attributes: hardness (kg), cohesiveness, and springiness (mm).

### 3.7. Microbiological Analysis

The total viable, mold, and yeast counts were determined using pour plate methods on plate count agar and potato dextrose agar, respectively, as described previously [36]. Briefly, 25 g of the cucumber samples were homogenized in sterile Ringer solution (225 mL) in a Stomacher blender for 1 min. Then, 10-fold serial dilutions were made by mixing 1 mL homogenate in 9 mL sterile Ringer solution. Once appropriate serial dilutions were achieved, the plating was carried out either on plate count agar or potato dextrose agar. The plates were incubated at 37 °C for 24–48 h for counting the total viable counts or at 25 °C for 2–7 days for counting the yeasts and molds, and the results were expressed as log10 cfu/g sample.

### 3.8. Determination of Total Phenolic Content (TPC) and DPPH Radical Scavenging Activity

Prior to analysis of the TPC and DPPH radical scavenging activity, a water extract of the cucumber samples was prepared by homogenizing 10 g of the sample in 100 mL distilled water, followed by filtration and centrifugation to obtain clear extract. For analysis of TPC, the Folin-Ciocalteu’s (FC) method was used as described previously [37]. Briefly, 0.5 mL extract was mixed with 0.5 mL of 1 M FC reagent and the mixture was incubated at room temperature for 5 min followed by addition of 1.5 mL of 1 M sodium carbonate, mixing and further incubation for 30 min at room temperature. After that, 2 mL of distilled water was added, and the absorbance of the sample and standard (0–200 mg/mL gallic acid) was measured at 725 nm using a Lambda EZ 150 spectrophotometer (PerkinElmer, Waltham, MA, USA); the results were specified as mg gallic acid equivalent (GAE)/g sample. The antiradical activity was determined by measuring the inhibition percentage of DPPH (2,2-Diphenyl-1-picrylhydrazyl) radicals by the cucumber extract as described by Singh et al. [38]. Briefly, 0.1 mL sample extract was diluted with 0.4 mL of Tris-HCl buffer (0.1 M, pH 7.4) and then 0.5 mL of 0.25 mM DPPH solution in methanol was added to the mixture, thoroughly mixed, and stored in the dark at room temperature for 30 min. The absorbance of the sample and the blank (without cucumber extract) was measured at 517 nm, and the percentage inhibition of DPPH radical was calculated using the following equation:

$$\mathrm{DPPH}\;\mathrm{radical}\;\mathrm{inhibition}\;\left(\%\right)=\frac{\mathrm{Absorbance}\;\mathrm{of}\;\mathrm{blank}-\mathrm{Absorbance}\;\mathrm{of}\;\mathrm{sample}}{\mathrm{Absorbance}\;\mathrm{of}\;\mathrm{blank}}\times 100$$

### 3.9. Statistical Analysis

The experiments were designed using a completely randomized block design with six treatments (A, B, C, D, E, and F), and measurements of the physicochemical quality attributes were taken at intervals of 0, 7, 14, and 21 days of cold storage. The entire blocks were independently replicated three times on three different days and the measurements of each quality attribute were taken in triplicate. The data obtained were subjected to analysis of variance (Two-way ANOVA) using SAS software (SAS Institute, Inc., Cary, NC, USA), and the means were compared using Duncan’s multiple range tests (DMRT) and the general linear model (GLM). Significance was accepted at *p* ≤ 0.05 and data were presented as means ± standard deviation (means ± SD) of three measurements. MULTBIPLOT software was used for the analysis of principal component analysis (PCA) and hierarchical cluster analysis (HCA) of data variables of the samples during storage intervals as described by Vicente-Villardón [39].

## 4. Conclusions

This study concludes that coating treatment and cold storage at 4 °C influenced the physicochemical, nutritional, and health quality attributes of cucumber at varied magnitudes. A functional coating with different concentrations of olive cake extract (OCE) and orange peel extract (OPE) increased the total soluble solids, hardness, total phenolic contents (TPC), and DPPH radical scavenging activity of the cucumber samples. However, prolonged storage (21 days) reduced total soluble solids, hardness, TPC, DPPH inhibition, and moisture content, and increased the total viable count (TVC), yeast and mold counts, and acidity of the product. Interestingly, functional coating alleviates the negative effect of storage on the above quality attributes. Overall, functional coating with OCE and OPE improved the physicochemical quality attributes and may extend the shelf-life of cucumber for up to 14 days of refrigerated storage.

## Figures and Tables

**Table 1:** Changes in water activity, moisture, and total soluble solids of fresh cucumber coated with chitosan and/or olive cake extract (OCE) or orange peel extract (OPE) during cold storage (4 °C).

Treatment	Storage Period (Days)			
	0	7	14	21
Moisture (%)				
A	93.24 ± 0.32 ^aB^	93.45 ± 0.17 ^aB^	91.79 ± 0.18 ^bB^	90.94± 0.25 ^cB^
B	94.30 ± 0.02 ^aA^	94.31 ± 0.47 ^aA^	92.65 ± 0.40 ^bA^	91.36 ± 0.39 ^cA^
C	94.09 ± 0.20 ^aA^	94.87 ± 0.28 ^bA^	93.08 ± 0.17 ^cA^	91.67 ± 0.23 ^dA^
D	94.54 ± 0.40 ^bA^	94.33 ± 0.27 ^aA^	93.02 ± 0.24 ^bA^	91.95 ± 0.19 ^cA^
E	94.25 ± 0.35 ^aA^	94.96 ± 0.30 ^aA^	93.23 ± 0.22 ^bA^	91.52 ± 0.28 ^cA^
F	94.95 ± 0.37 ^aA^	94.44 ± 0.27 ^aA^	92.99 ± 0.81 ^bA^	91.57 ± 0.42 ^cA^
Water activity (aw)				
A	0.993 ± 0.011	0.990 ± 0.007	0.985 ± 0.006	0.978 ± 0.009
B	0.992 ± 0.005	0.990 ± 0.009	0.986 ± 0.008	0.980 ± 0.011
C	0.992 ± 0.006	0.990 ± 0.004	0.985 ± 0.007	0.979 ± 0.004
D	0.992 ± 0.009	0.990 ± 0.001	0.984 ± 0.017	0.979 ± 0.009
E	0.992 ± 0.006	0.990 ± 0.007	0.985 ± 0.005	0.979 ± 0.013
F	0.992 ± 0.002	0.990 ± 0.009	0.984 ± 0.004	0.980 ± 0.001
Total soluble solids (%)				
A	4.60 ± 0.09 ^aB^	4.00 ± 0.20 ^bB^	3.20 ± 0.20 ^cB^	2.4 ± 0.20 ^dB^
B	4.80 ± 0.10 ^aB^	4.20 ± 0.19 ^bB^	3.40 ± 0.09 ^cB^	2.6 ± 0.19 ^dB^
C	5.00 ± 0.19 ^aA^	4.60 ± 0.14 ^bA^	3.80 ± 0.14 ^cA^	3.0 ± 0.34 ^dA^
D	5.20 ± 0.18 ^aA^	4.80 ± 0.13 ^bA^	4.00 ± 0.13 ^cA^	3.2 ± 0.33 ^dA^
E	5.20 ± 0.20 ^aA^	4.60 ± 0.19 ^bA^	3.80 ± 0.19 ^cA^	3.0 ± 0.19 ^dA^
F	5.00 ± 0.19 ^aA^	4.60 ± 0.24 ^bA^	3.80 ± 0.14 ^cA^	3.0 ± 0.34 ^dA^

Values are means of triplicate samples (±SD). Means not sharing common lower-case letters in a row (storage) or capital letters in a column (treatments) are significantly different at *p* ≤ 0.05 as assessed by Duncan’s Multiple Range Test. A: Uncoated, B: Coated with 2% chitosan only, C: Coated with 2% chitosan containing 1% OCE, D: Coated with 2% chitosan containing 2% OCE, E: Coated with 2% chitosan containing 1% OPE, and F: Coated with 2% chitosan containing 2% OPE.

**Table 2:** Changes in pH, acidity, total phenolics, and antioxidant activity of fresh cucumber coated with chitosan and/or olive cake extract (OCE) or orange peel extract (OPE) during cold storage (4 °C).

Treatment	Storage Period (Days)			
	0	7	14	21
pH				
A	5.86 ± 0.03 ^aB^	5.70 ± 0.02 ^bE^	5.25 ± 0.22 ^cD^	4.03 ± 0.01 ^dE^
B	5.79 ± 0.01 ^aC^	5.80 ± 0.00 ^aD^	5.92 ± 0.20 ^aC^	5.18 ± 0.01 ^bD^
C	5.85 ± 0.04 ^bB^	5.97 ± 0.02 ^aC^	6.23 ± 0.25 ^aB^	5.28 ± 0.01 ^cC^
D	5.87 ± 0.03 ^cAB^	5.98 ± 0.00 ^bC^	6.29 ± 0.20 ^aB^	5.25 ± 0.02 ^dC^
E	5.86 ± 0.01 ^cB^	6.10 ± 0.02 ^bB^	6.68 ± 0.18 ^aA^	5.62 ± 0.01 ^dB^
F	5.90 ± 0.00 ^cA^	6.26 ± 0.05 ^bA^	6.81 ± 0.15 ^aA^	5.72 ± 0.01 ^dA^
Titratable acidity (% malic acid)				
A	0.78 ± 0.01 ^dA^	0.83 ± 0.03 ^cA^	1.02 ± 0.01 ^bA^	1.24 ± 0.01 ^aA^
B	0.77 ± 0.01 ^dA^	0.79 ± 0.03 ^cA^	0.99 ± 0.01 ^bB^	1.22 ± 0.02 ^aA^
C	0.77 ± 0.01 ^cA^	0.79 ± 0.02 ^cA^	0.98 ± 0.01 ^bB^	1.22 ± 0.01 ^aA^
D	0.77 ± 0.01 ^cA^	0.78 ± 0.01 ^cA^	0.97 ± 0.01 ^bB^	1.19 ± 0.02 ^aB^
E	0.76 ± 0.01 ^cA^	0.78 ± 0.01 ^cA^	0.97 ± 0.03 ^bB^	1.18 ± 0.03 ^aB^
F	0.74 ± 0.01 ^cB^	0.75 ± 0.01 ^cB^	0.93 ± 0.03 ^bC^	1.15 ± 0.01 ^aC^
Total phenolics (mg GAE/g)				
A	18.82 ± 0.26 ^aE^	16.25 ± 0.36 ^bE^	11.13 ± 0.49 ^cE^	3.79 ± 0.45 ^dE^
B	19.74 ± 0.28 ^aD^	17.44 ± 0.36 ^bD^	12.33 ± 0.37 ^cD^	4.95 ± 0.34 ^dD^
C	21.05 ± 0.27 ^aC^	18.73 ± 0.37 ^bC^	13.72 ± 0.43 ^cC^	6.38 ± 0.43 ^dC^
D	23.92 ± 0.23 ^aB^	19.29 ± 0.39 ^bC^	14.19 ± 0.39 ^cC^	6.85 ± 0.36 ^dC^
E	23.40 ± 0.16 ^aB^	20.15 ± 0.37 ^bB^	15.33 ± 0.41 ^cB^	7.99 ± 0.41 ^dB^
F	25.87 ± 0.38 ^aA^	22.25 ± 0.37 ^bA^	17.31 ± 0.66 ^cA^	9.98 ± 0.12 ^dA^
DPPH inhibition (%)				
A	24.66 ± 0.17 ^bE^	22.05 ± 0.39 ^cE^	29.29 ± 0.70 ^aE^	17.34 ± 0.70 ^dE^
B	22.56 ± 0.83 ^bF^	19.95 ± 1.02 ^cF^	27.53 ± 0.17 ^aF^	15.57 ± 0.10 ^dF^
C	28.70 ± 0.39 ^bD^	26.09 ± 0.45 ^cD^	35.61 ± 0.42 ^aD^	23.65 ± 0.42 ^dD^
D	39.65 ± 0.17 ^bC^	37.04 ± 0.39 ^cC^	38.60 ± 0.19 ^aC^	26.65 ± 0.19 ^dC^
E	49.75 ± 0.54 ^aB^	47.22 ± 0.45 ^bB^	43.22 ± 0.70 ^cB^	31.26 ± 0.70 ^dB^
F	57.32 ± 0.99 ^bA^	54.71 ± 0.85 ^cA^	62.54 ± 1.56 ^aA^	50.59 ± 1.61 ^dA^

Values are means of triplicate samples (±SD). Means not sharing common lower-case letters in a row (storage) or capital letters in a column (treatments) are significantly different at *p* ≤ 0.05 as assessed by Duncan’s Multiple Range Test. A: Uncoated, B: Coated with 2% chitosan only, C: Coated with 2% chitosan containing 1% OCE, D: Coated with 2% chitosan containing 2% OCE, E: Coated with 2% chitosan containing 1% OPE, and F: Coated with 2% chitosan containing 2% OPE.

**Table 3:** Changes in surface color of fresh cucumber coated with chitosan and/or olive cake extract (OCE) or orange peel extract (OPE) during cold storage (4 °C).

Treatment	Storage Period (Days)			
	0	7	14	21
L*				
A	48.25 ± 1.09 ^aA^	47.26 ± 0.13 ^bA^	44.12 ± 0.59 ^cA^	43.05 ± 0.77 ^dA^
B	43.82 ± 0.67 ^aB^	42.91 ± 0.18 ^bB^	42.37 ± 0.75 ^bB^	38.54 ± 0.21 ^cC^
C	40.49 ± 0.39 ^aC^	38.38 ± 0.90 ^bD^	38.88 ± 0.13 ^bD^	33.04 ± 0.34 ^cD^
D	43.92 ± 0.51 ^aB^	39.69 ± 0.28 ^cC^	41.98 ± 0.06 ^bC^	37.45 ± 0.57 ^dC^
E	43.40 ± 0.26 ^aB^	39.32 ± 0.02 ^cC^	42.11 ± 0.90 ^bB^	38.30 ± 0.58 ^dC^
F	43.26 ± 0.94 ^aB^	41.62 ± 0.61 ^bB^	41.48 ± 0.13 ^bC^	40.25 ± 0.39 ^cB^
a*				
A	−16.24 ± 0.78 ^cA^	−15.69 ± 0.46 ^bB^	−13.93 ± 0.66 ^bC^	−11.55 ± 0.73 ^aE^
B	−14.79 ± 0.42 ^bC^	−14.24 ± 0.15 ^aC^	−14.31 ± 0.31 ^bB^	−14.60 ± 0.34 ^bC^
C	−13.80 ± 0.24 ^bD^	−17.63 ± 0.41 ^dA^	−12.71 ± 0.86 ^aD^	−14.68 ± 0.55 ^cC^
D	−15.83 ± 0.67 ^cB^	−15.70 ± 0.19 ^cB^	−14.00 ± 0.34 ^bB^	−13.20 ± 0.63 ^aD^
E	−14.25 ± 0.80 ^aC^	−14.71 ± 0.65 ^aC^	−15.32 ± 0.26 ^bA^	−16.10 ± 0.06 ^cA^
F	−5.48 ± 0.25 ^aE^	−13.66 ± 0.26 ^bD^	−13.26 ± 0.99 ^bC^	−15.14 ± 0.20 ^cB^
b*				
A	23.21 ± 0.09 ^aA^	18.53 ± 0.05 ^bE^	18.08 ± 0.08 ^cB^	15.97 ± 0.19 ^dF^
B	19.29 ± 0.14 ^bC^	21.69 ± 0.07 ^aC^	18.17 ± 0.16 ^cB^	18.37 ± 0.28 ^cD^
C	17.27 ± 0.12 ^cE^	17.83 ± 0.15 ^aF^	15.91 ± 0.04 ^dD^	19.62 ± 0.07 ^bB^
D	21.46 ± 0.05 ^aB^	19.69 ± 0.13 ^bD^	18.00 ± 0.18 ^cB^	17.93 ± 0.18 ^dE^
E	18.73 ± 0.16 ^cD^	23.83 ± 0.15 ^dA^	20.84 ± 0.19 ^bA^	21.11 ± 0.98 ^aA^
F	18.99 ± 0.13 ^cD^	24.03 ± 0.16 ^aA^	21.74 ± 0.18 ^dA^	20.48 ± 0.15 ^bA^

Values are means of triplicate samples (±SD). Means not sharing common lower-case letters in a row (storage) or capital letters in a column (treatments) are significantly different at *p* ≤ 0.05 as assessed by Duncan’s Multiple Range Test. A: Uncoated, B: Coated with 2% chitosan only, C: Coated with 2% chitosan containing 1% OCE, D: Coated with 2% chitosan containing 2% OCE, E: Coated with 2% chitosan containing 1% OPE, and F: Coated with 2% chitosan containing 2% OPE.

**Table 4:** Changes in texture profile of fresh cucumber coated with chitosan and/or olive cake extract (OCE) or orange peel extract (OPE) during cold storage (4 °C).

Treatment	Storage Period (Days)			
	0	7	14	21
Hardness (kg)				
A	1181.33 ± 1.25 ^aD^	1084.17 ± 0.45 ^bB^	880.33 ± 0.76 ^cB^	660.50 ± 0.44 ^dC^
B	1204.00 ± 1.09 ^aA^	1072.00 ± 1.77 ^bD^	855.67 ± 0.88 ^cE^	667.33 ± 0.32 ^dB^
C	1151.83 ± 1.45 ^aE^	1063.00 ± 2.98 ^bF^	861.00 ± 1.22 ^cD^	674.50 ± 0.87 ^dA^
D	1180.67 ± 0.33 ^aD^	1079.33 ± 1.35 ^bC^	891.67 ± 1.19 ^cA^	675.50 ± 0.29 ^dA^
E	1183.33 ± 0.29 ^aC^	1099.00 ± 0.48 ^bA^	868.00 ± 0.99 ^cC^	657.33 ± 0.03 ^dD^
F	1199.33 ± 2.69 ^aB^	1068.17 ± 1.06 ^bE^	857.50 ± 1.21 ^cE^	626.67 ± 0.05 ^dE^
Cohesiveness				
A	0.85 ± 0.01 ^a^	0.81 ± 0.02 ^b^	0.81 ± 0.01 ^b^	0.82 ± 0.00 ^b^
B	0.85 ± 0.02 ^a^	0.84 ± 0.04 ^a^	0.81 ± 0.02 ^b^	0.82 ± 0.01 ^b^
C	0.85 ± 0.01 ^a^	0.81 ± 0.06 ^a^	0.81 ± 0.02 ^b^	0.82 ± 0.01 ^b^
D	0.85 ± 0.02 ^a^	0.82 ± 0.01 ^b^	0.81 ± 0.01 ^b^	0.81 ± 0.01 ^b^
E	0.84 ± 0.00 ^a^	0.81 ± 0.02 ^b^	0.82 ± 0.03 ^a^	0.82 ± 0.02 ^a^
F	0.85 ± 0.02 ^a^	0.81 ± 0.02 ^b^	0.83 ± 0.01 ^a^	0.81 ± 0.01 ^b^
Springiness (mm)				
A	0.87 ± 0.02 ^a^	0.87 ± 0.01 ^a^	0.90 ± 0.04 ^a^	0.83 ± 0.01 ^b^
B	0.90 ± 0.01	0.90 ± 0.03	0.90 ± 0.02	0.87 ± 0.01
C	0.90 ± 0.00 ^a^	0.90 ± 0.02 ^a^	0.87 ± 0.03 ^a^	0.80 ± 0.01 ^b^
D	0.90 ± 0.01	0.90 ± 0.05	0.90 ± 0.01	0.87 ± 0.03
E	0.90 ± 0.04	0.90 ± 0.01	0.90 ± 0.01	0.90 ± 0.02
F	0.90 ± 0.03 ^a^	0.90 ± 0.02 ^a^	0.90 ± 0.03 ^a^	0.83 ± 0.01 ^b^

Values are means of triplicate samples (±SD). Means not sharing common lower-case letters in a row (storage) or capital letters in a column (treatments) are significantly different at *p* ≤ 0.05 as assessed by Duncan’s Multiple Range Test. A: Uncoated, B: Coated with 2% chitosan only, C: Coated with 2% chitosan containing 1% OCE, D: Coated with 2% chitosan containing 2% OCE, E: Coated with 2% chitosan containing 1% OPE, and F: Coated with 2% chitosan containing 2% OPE.

## Data Availability

The data presented in this study are available in this article.
